# Supplementary figures and images for: Peanut-based intercropping systems altered soil bacterial communities, potential functions, and crop yield
Source: PeerJ. 2024 Feb 7;12:e16907. doi: 10.7717/peerj.16907 (PMC10858685; doi:10.7717/peerj.16907)

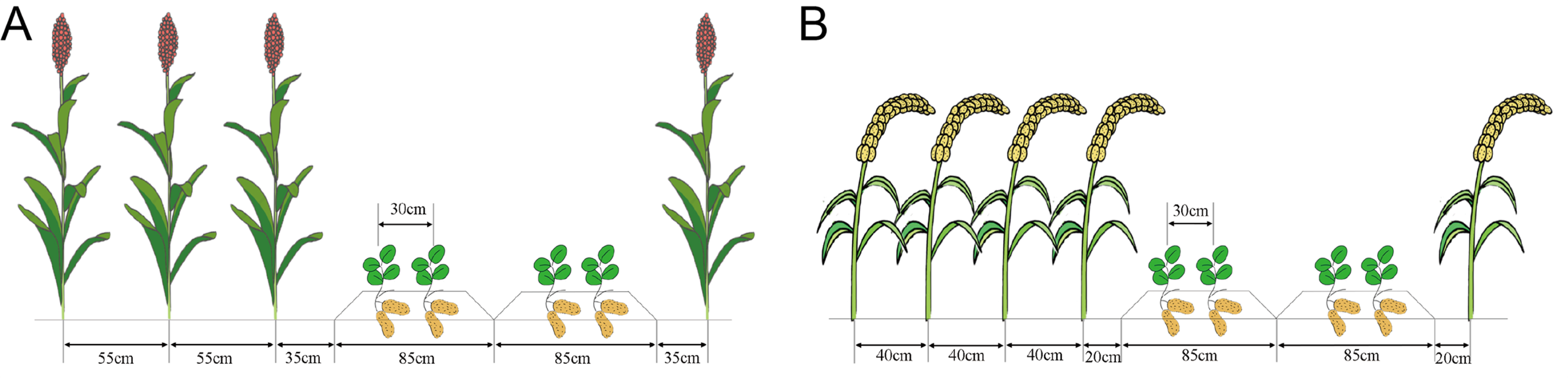

Supplement: Figure S1 [file peerj-12-16907-s001.png]

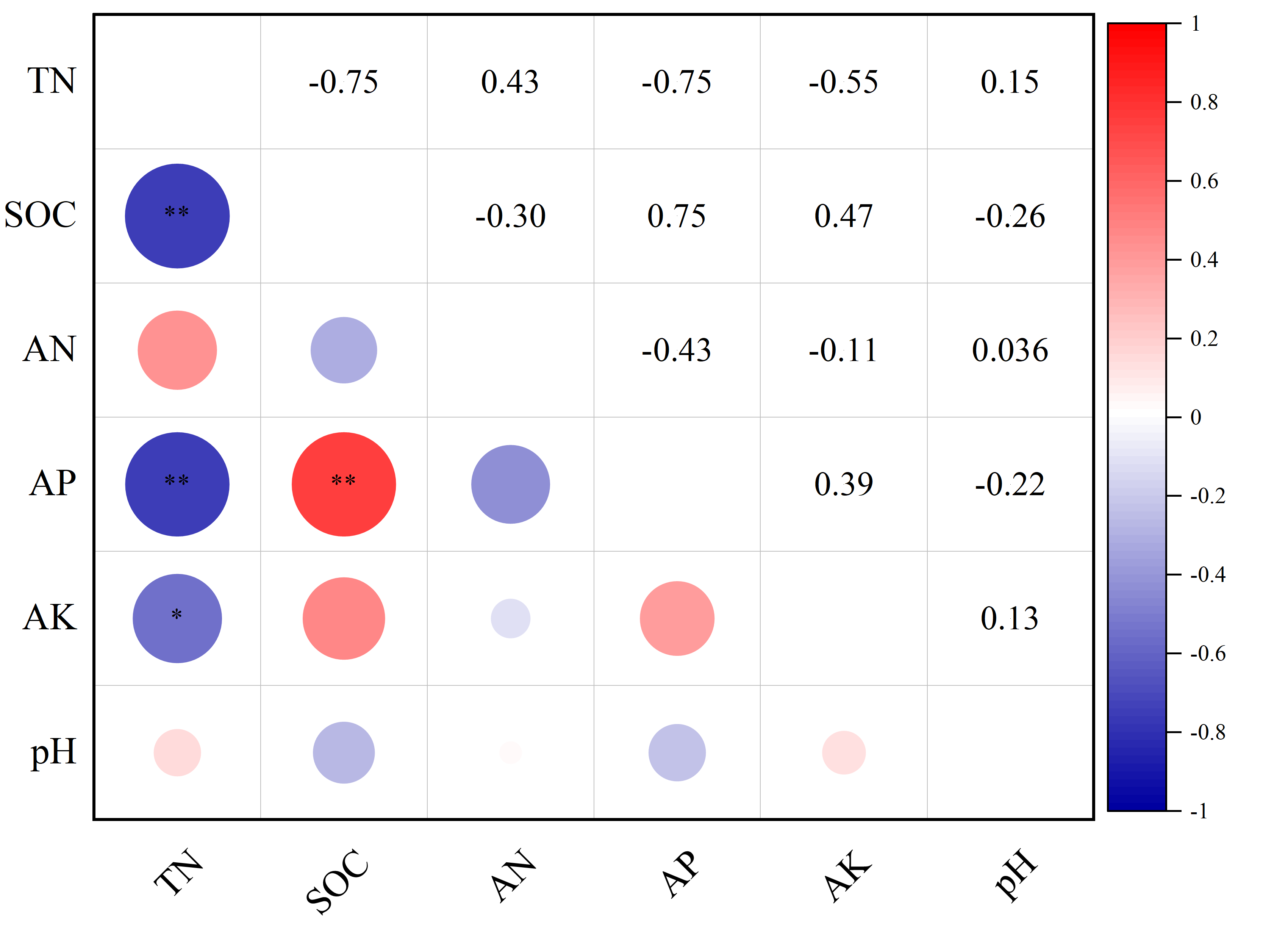

Supplement: Figure S2 — *p < 0.05, **p < 0.01, ***p < 0.001. [file peerj-12-16907-s002.png]

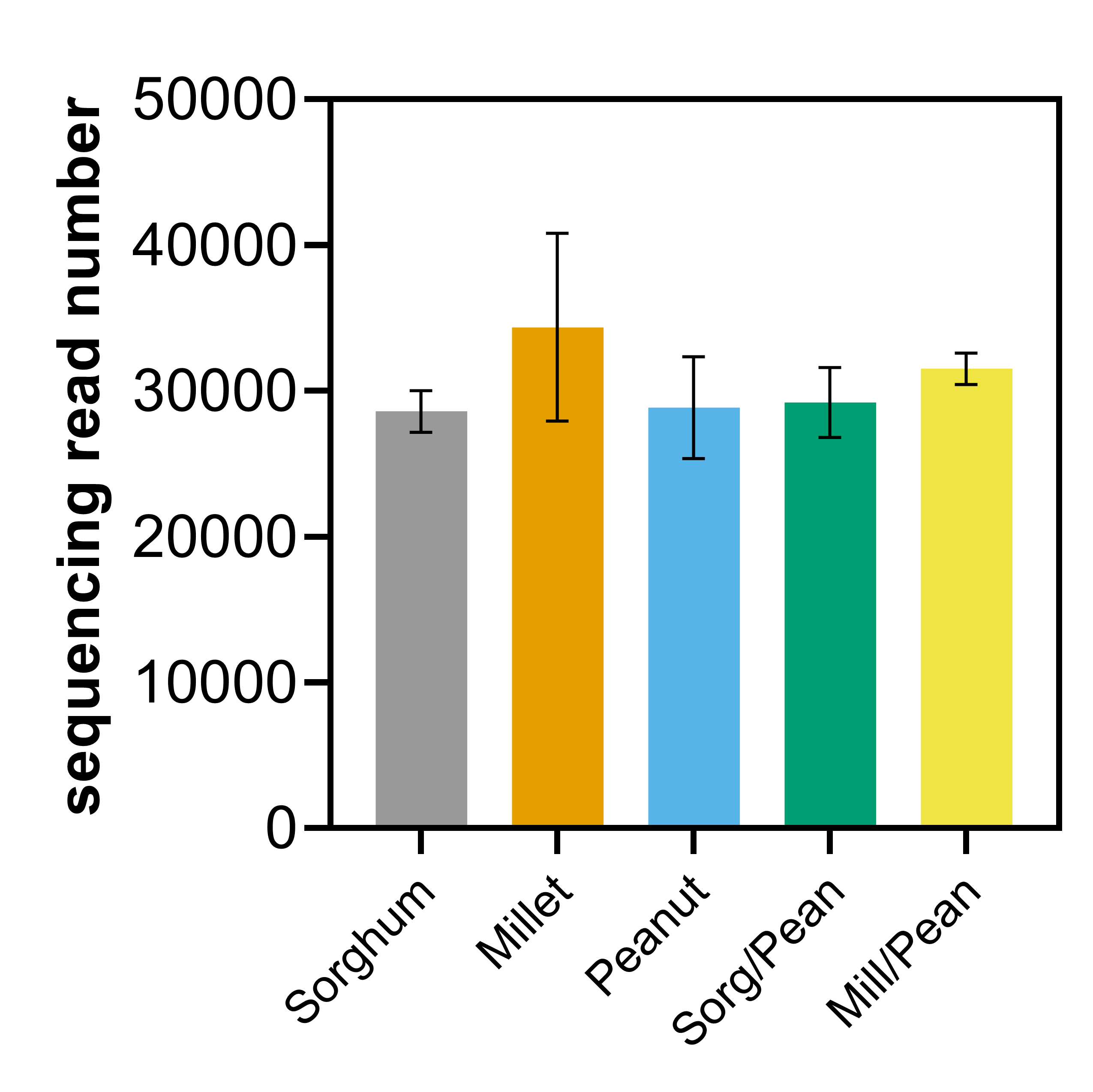

Supplement: Figure S3 [file peerj-12-16907-s003.png]

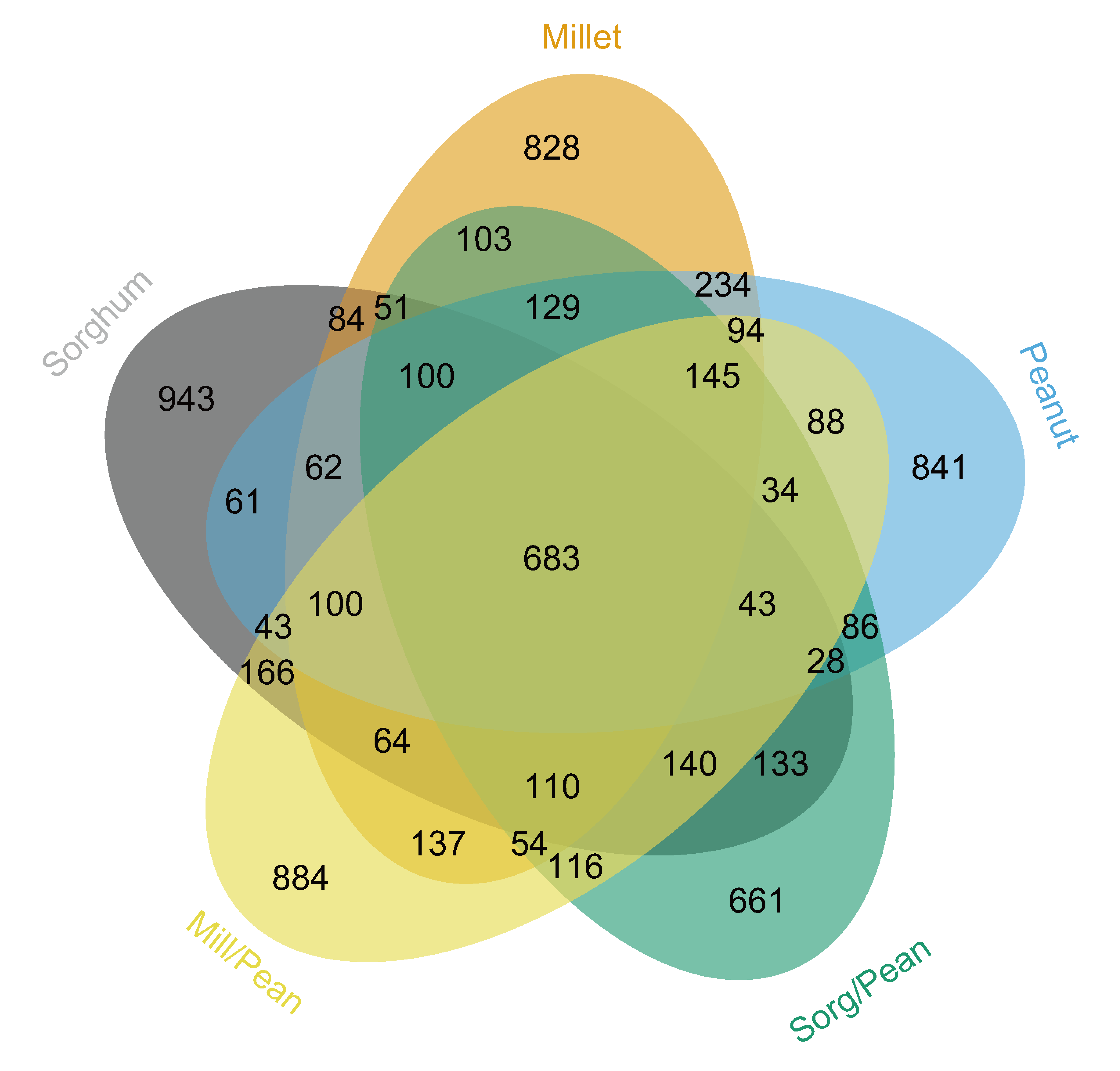

Supplement: Figure S4 [file peerj-12-16907-s004.png]

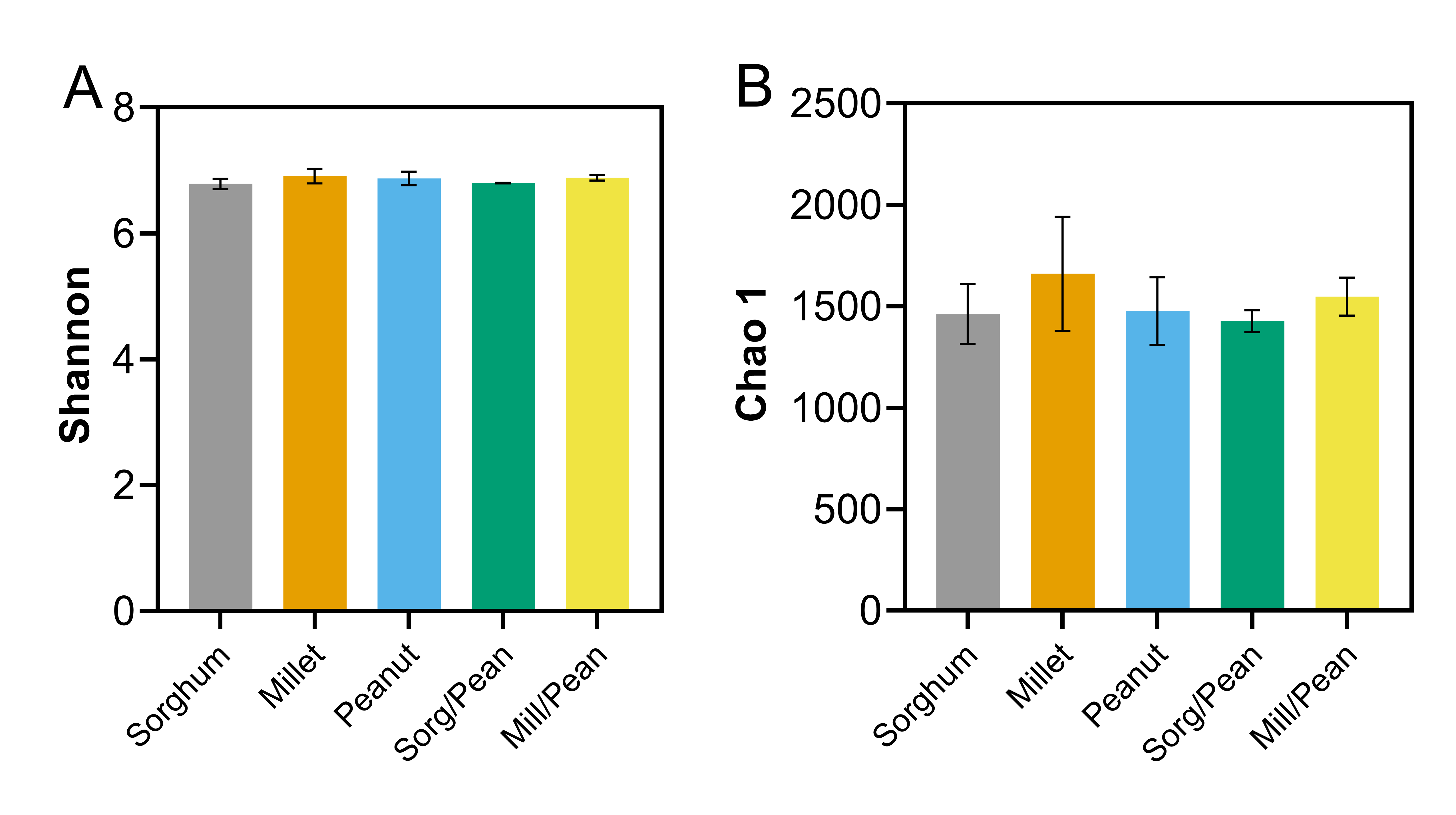

Supplement: Figure S5 — (A) Shannon index; (B) chao1 index. [file peerj-12-16907-s005.png]

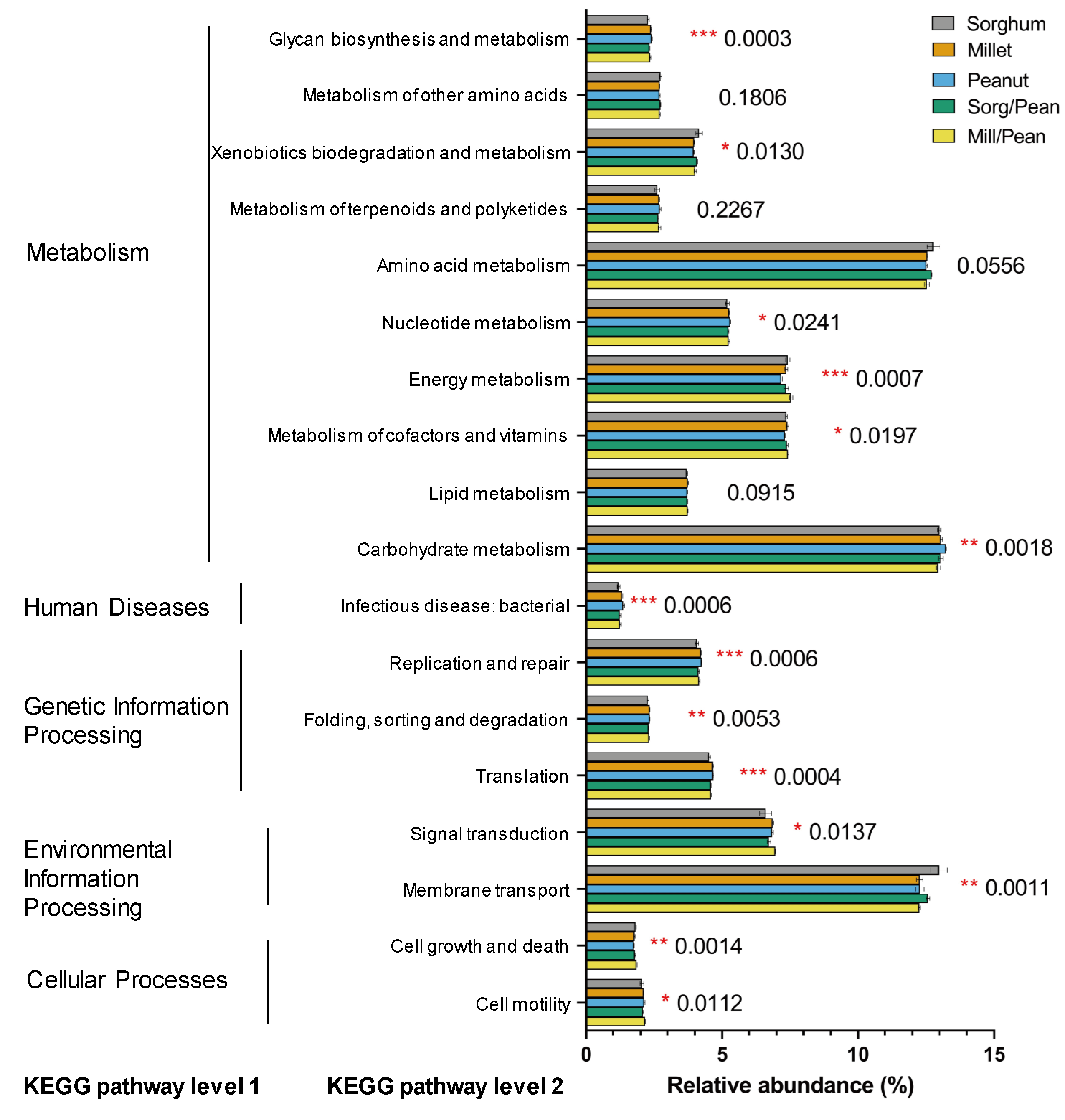

Supplement: Figure S6 — *p < 0.05, **p < 0.01, ***p < 0.001. [file peerj-12-16907-s006.png]
